# The MATCHIT Automaton: Exploiting Compartmentalization for the Synthesis of Branched Polymers

**DOI:** 10.1155/2013/467428

**Published:** 2013-12-31

**Authors:** Mathias S. Weyland, Harold Fellermann, Maik Hadorn, Daniel Sorek, Doron Lancet, Steen Rasmussen, Rudolf M. Füchslin

**Affiliations:** ^1^European Centre for Living Technology, S. Marco 2940, 30124 Venice, Italy; ^2^Center for Fundamental Living Technology (FLinT), Department of Physics, Chemistry and Pharmacy, University of Southern Denmark, 5230 Odense, Denmark; ^3^Department of Chemistry and Applied Biosciences, ETH Zurich, 8093 Zurich, Switzerland; ^4^The Lancet Lab, Department of Molecular Genetics, Weizmann Institute of Science, 76100 Rehovot, Israel; ^5^Santa Fe Institute, Santa Fe, NM 87501, USA; ^6^Institute of Applied Mathematics and Physics, School of Engineering, Zurich University of Applied Sciences, 8401 Winterthur, Switzerland

## Abstract

We propose an automaton, a theoretical framework that demonstrates how to improve the yield of the synthesis of branched chemical polymer reactions. This is achieved by separating substeps of the path of synthesis into compartments. We use chemical containers (chemtainers) to carry the substances through a sequence of fixed successive compartments. We describe the automaton in mathematical terms and show how it can be configured automatically in order to synthesize a given branched polymer target. The algorithm we present finds an optimal path of synthesis in linear time. We discuss how the automaton models compartmentalized structures found in cells, such as the endoplasmic reticulum and the Golgi apparatus, and we show how this compartmentalization can be exploited for the synthesis of branched polymers such as oligosaccharides. Lastly, we show examples of artificial branched polymers and discuss how the automaton can be configured to synthesize them with maximal yield.

## 1. Introduction

Recently, small scale personal manufacturing has seen a rapid increase in popularity with emerging technologies such as 3D printing. In place of central production and physical distribution of goods, personal manufacturing offers a transfer of information (e.g., designs, protocols) followed by in-place, customizable production. Although mainly discussed in the context of macroscale personal manufacturing (i.e., 3D printing), personal manufacturing is also found in the domain of (bio-)chemistry. In custom oligonucleotide and peptide synthesis, the information, that is, DNA or protein sequence, is sent by the customer to the supplier. Even though the synthesized DNA oligonucleotides or peptides are shipped back to the customer (i.e., transfer of goods), this example demonstrates how transferred information can lead to in-place, customizable production of (bio)chemical goods. However, often the desired product cannot be synthesized via either one-pot synthesis or sequential one-pot synthesis. Consequently, distinct confinement and transport of substances and an elaborate temporal and spatial reaction management are needed.

The European Commission funded project MATCHIT (Matrix for Chemical IT [1, 2]) aims to open the domain of chemistry for distributed manufacturing by implementing unconventional embedded computation systems. By employing addressable soft colloid supermolecular chemical containers (chemtainers) that act both as transport and reaction vessels and that are interfaced with electronic computers via microelectromechanical systems (MEMS), the topological organization of the cytoplasm of natural cells is mimicked, where a multitude of chemicals is organized in addressable compartments and where transport and fusion of compartments trigger reactions of previously separated chemicals. By utilizing a hybrid biochemical and information technological system, an integrated programmable material transportation, information processing and material production system was implemented resulting in a generic programmable platform for complex molecular processing tasks.

In contrast to other approaches that either employ “traditional” 3D printing techniques to implement small scale personal manufacturing in (bio)chemistry [3] or use of simpler building blocks like lipid-coated aqueous droplets in oil [4], in the MATCHIT machine, DNA oligonucleotides attached to the surface of the chemtainers (DNA addresses) are employed to guide the chemtainer's assembly, fusion promoting chemical reactions, and reversible docking on defined spots of the microfluidic environment through reversible hybridization. The DNA addresses thereby dynamically change and adapt as a result of ongoing DNA-computation operations such as relabeling and simple Boolean operations [5–7]; the current set of DNA addresses therefore both portrays the chemtainers history and defines the next steps to take (e.g., with whom to bind and fuse, where to dock). In this context, the MEMS technology is perfectly suited not only for precise and accurate guidance the chemtainers creation [8], loading, and manipulation but also for an on-chip pre- and postprocessing (e.g., electrophoretic separation) of substances released from the chemtainers or waiting for reencapsulation.

The modular design of the MATCHIT machine allowed for an individual testing and optimization of each of its components (i.e., chemtainers, MEMS, and computer science). In the context of the chemtainers, we reported the first DNA-computation operations on the surface of artificial giant unilamellar vesicles (GUVs), DNA-mediated self-assembly [9, 10] and controlled fusion [11–13] of GUVs and oil-in-water droplets, and increase in the complexity of available soft colloid chemtainers by preparing hierarchically organized GUVs [14]. The research efforts of integrating chemical and electronic systems in a MEMS device resulted in unconventional embedded computation systems that performed complex nanoscale chemical tasks autonomously [15]. Traditional computer science was not only used for programing the MEMS devices. By using the same language for simulating the inherent complexity of MATCHIT objects and operations and to program the MEMS devices, solutions found *in silico* were directly transferred to control the MEMS devices. In this context, the MATCHIT automaton (MA) is able to optimize the microfluidics design in terms of structure, MEMS and chemtainer DNA addresses, chemtainer types, and interaction rules.

Our motivation to demonstrate how the MA can be applied to the synthesis of branched polymers (e.g., branched oligosaccharides) is as follows: often oligosaccharides are attached to proteins and impart an additional level of information content to the underlying protein structures [16]. The glycosylation is a critical function of the biosynthetic-secretory pathway in the endoplasmic reticulum (ER) and the Golgi apparatus. Glycoproteins associated with cell surface and intracellular proteins have crucial biological and physiological roles, from contributions in protein folding and quality control to involvement in a large number of biological recognition events [16, 17]. In the ER, for example, glycosylation is used to monitor the status of protein folding, acting as a quality control mechanism to ensure that only proteins properly folded are trafficked to the Golgi. Oligosaccharides are composed of monosaccharides each having four to five binding sites. This large number of binding sites and the vast number of distinct monosaccharides constitute the large variety of oligosaccharides. Thus, the glycan structures attached to proteins can be highly complex, with numerous possibilities for branching and anomeric linkage. Consequently, glycoproteins have much greater structural diversity than linear nucleic or polypeptide structures. Hence, the custom synthesis of such complex branched molecules is a demanding task because the control of potential side reactions is still not solved [18]. In nature, glycosylation occurs in a stepwise fashion by trafficking glycoproteins to distinct spatially separated compartments (i.e., Golgi cisternae) that contain a distinct set of enzymes.

One approach to tackle the problem of side reactions is to use linker structures to increase the yield in one-pot reactions. Such small linker structures have been implemented and are known by the term click chemistries. The concept of click chemistries is for example, described in Kolb et al. [19–21] and addresses powerful, highly reliable, and selective reactions for the rapid synthesis of new compounds through heteroatom links. In the context of oligosaccharide synthesis however, the number of such click-chemistry linkers is highly limited, leading to a significant, but moderate, increase of yield [22]. Hence, our approach is to augment the effect of these small linker structures by means of additional compartmentalization. Specifically, the substances (e.g., oligosaccharide) are enclosed by chemtainers which can be transported between compartments and which can be addressed by tags.

In this paper, we study an artificial chemistry [23] that models the synthesis of branched polymers. The focus of this work lies in the description of a compiler for a defined reaction environment, the MA. By a compiler we understand a method that takes the description of a branched oligomer as input and maps it onto system parameters of the MA such that this automaton synthesizes the input structure. We refer to these system parameters as the configuration of the MA. With such a compiler, we present an example of what is often called ChemBio-IT [24], the merger of computer science and (bio)chemistry. The MA offers a platform with which such combinatorial variety of different types of branched oligomers—a promising tool in a future personalized medicine—can be generated. This work presents a mathematical formalism to encode a large and biologically important class of such oligomers and thereby enables automatized generation of protocols for the synthesis of specific structures.

The contribution of this work is twofold. First, we present a mathematical framework that describes the MA. Second, we discuss an algorithm—the compiler—to configure the MA in order to synthesize branched polymers. The remainder of this paper is structured as follows. In Section 2, we describe the MA in mathematical terms (Section 2.1), we suggest how branched polymers can be encoded into a mathematical graph structure (Section 2.2), and we describe how the MA configuration can be derived from a given target polymer (Section 2.3). In Section 3, we discuss some results obtained using these methods. We compare the MA to another artificial reactor and we propose improvements for the shortcomings of the MA. We finish this paper with a conclusion in Section 4 where we hypothesize how real instances of the MA could be used in the future.

## 2. Methods

### 2.1. Description of the MATCHIT Automaton

The MA can be seen as a model of the spatially separated compartments of the ER and Golgi that contain distinct sets of enzymes. In particular, the MA consists of the following entities. (a) *Substances.* That is, monomers, polymers or a mixture thereof. (b) *Reactions.* That is, translation rules that determine how two substances react into a third substance (cf. enzymes). (c) *Chemtainers.* That is, compartments containing a substance and equipped with one or more chemtainer tags. (d) *Cells.* That is, compartments containing chemtainers, equipped with a cell tag. (e) *Tags.* That is, a mechanism that induces chemtainers to stick to cells (chemtainer cell interaction) or to each other (chemtainer chemtainer interaction). (f) *Tube.* That is, a chain of concatenated cells. (g) *Inlet.* That is, the mechanism to stage chemtainers and insert them into the tube.

**Figure 1:**
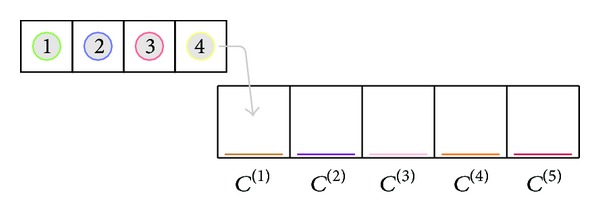
Overview of the elements of the MATCHIT automaton. The inlet is populated with four chemtainers, carrying the substances 1 to 4, respectively, and equipped with different chemtainer tags (color of circle). The tube consists of five cells which are equipped with individual cell tags. The color of the bar at the bottom of each cell implies which particular cell tag is used. The arrow shows how chemtainer at the front of the inlet would be inserted into the tube.

The MA operates in a discretized time domain.  Figure 1 shows an overview of the MA and its entities. The general working principle is as follows. Chemtainers containing substances are staged inside an inlet and inserted into the tube consisting of various cells. The chemtainers move from cell to cell and can fuse under some conditions controlled by a tag system. In the event of a chemtainer fusion, the substances carried inside the fused chemtainers mix and can react with each other. The product of such a reaction remains inside the chemtainer, but this chemtainer can fuse with other chemtainers in subsequent steps. As we will demonstrate, this allows for a very granular control of the sequence of reaction steps contained inside the chemtainers. The following sections describe the various aspects of the system in more detail.

#### 2.1.1. Inlet, Tube, and Cells

The tube is a list of *m* cells *C* ^(1)^,…, *C* ^(*m*)^. The movement of chemtainers from cell to cell is governed by the following rules. (a) Chemtainers from the inlet are always inserted into *C* ^(1)^. (b) Chemtainers can only move from *C* ^(*j*)^ to *C* ^(*j*+1)^. (c) Chemtainers in *C* ^(*m*)^ remain there (end of the tube).

The inlet is a list of chemtainers; its purpose is to insert chemtainers into the tube. There is only one inlet and it is inserting chemtainers into the first cell of the tube, that is, *C* ^(1)^.

#### 2.1.2. Chemtainer

A chemtainer *c*_*i*_ is a tuple (*s*_*i*_, *T*_*i*_, *μ*_*i*_) where *s*_*i*_ ∈ *S* is a substance and *T*_*i*_ ⊂ *T* ^Chem^ is a set of tags assigned to the chemtainer. Each chemtainer maintains a counter *μ*_*i*_ which tells how many time steps are left for the chemtainer to stick to the current cell before moving to the next cell. This allows for colocation and subsequent fusion of chemtainers.

#### 2.1.3. Substance and Reaction

Exactly two substances can react with each other; the result of a reaction is a product substance. The notation for such a reaction is (*s*_*i*_ + *s*_*j*_ → *s*_*p*_) where *s*_*i*_ and *s*_*j*_ react to the product *s*_*p*_. Note that a reaction exists for any pair *s*_*i*_ and *s*_*j*_; if the reactants cannot react chemically, the resulting product is a mixture of *s*_*i*_ and *s*_*j*_ which is considered to be waste. *S* denotes the set of all substances and *S*′ ⊂ *S* is the set of all monomers.

#### 2.1.4. Tag and Affinity

Tags are used to determine whether chemtainers can interact with each other and whether they can stick to cells. There are two kinds of tags: chemtainer-tags *t* ^Chem^ ∈ *T* ^Chem^ and cell tags *t* ^Cell^ ∈ *T* ^Cell^. The former are a property of chemtainers; the latter are a property of cells.

For *T* = *T* ^Chem^ ∪ *T* ^Cell^ there is an affinity function *a* : *T* × *T* → *ℕ*_0_ which assigns a nonnegative integer to each combination of tags *t*_*i*_ and *t*_*j*_ such that *a*(*t*_*i*_, *t*_*j*_) = *a*(*t*_*j*_, *t*_*i*_). This affinity *a* between two tags is interpreted as follows. (a) If *t*_*i*_ ∈ *T* ^Chem^ and *t*_*j*_ ∈ *T* ^Cell^, *a* indicates the total number of time steps the chemtainer takes to stick to the cell. *a* = 0 means that the chemtainer does not stick to the cell at all. (b) If *t*_*i*_, *t*_*j*_ ∈ *T* ^Chem^, *a* > 0 indicates that two chemtainers can potentially fuse, and *a* = 0 indicates that two chemtainers cannot fuse. (c) If *t*_*i*_, *t*_*j*_ ∈ *T* ^Cell^, *a* has no meaning since the cells are static and cell tags never interact with each other. Hence this affinity is not defined.

#### 2.1.5. Dynamics

The time domain is discretized into time steps *τ* ∈ *ℕ*_0_. At time step *τ* = 0, the inlet is filled with *n* chemtainers and no chemtainer has moved or reacted yet.

Starting from time step *τ* = 1 onwards, the following steps are executed in the order stated. (a) Move chemtainers from one cell to the next: the counter *μ*_*i*_ of each chemtainer *c*_*i*_ is decreased by 1. If *μ*_*i*_ = 0 before decreasing, *c*_*i*_ is moved to the next cell. The chemtainer counter *μ*_*i*_ is reset according to the largest chemtainer cell affinity; that is,

(1) μi=max⁡tj(a(tj,tc))

over all the chemtainer tags *t*_*j*_ of chemtainer *c*_*i*_; *t*_*c*_ is the chemtainer tag of the next cell. (b) Move one chemtainer from the front of the inlet to the first cell in the tube *C* ^(1)^. (c) Chemtainer fusion and reactions: for all chemtainers that are both sticking to the same cell *j* (i.e., with *μ*_*i*_ > 0) and sticking together by the means of chemtainer chemtainer interaction, fuse the two chemtainers to a single one and apply the appropriate reaction to the chemtainer contents. Note that a cell may potentially contain more than two chemtainers, in which case the order of fusion has to be defined. The algorithm described below (see Section 2.3.3) however ensures that such a situation never occurs; hence we do not define the order of fusion for such a case.

After the consecutive execution of these three steps, *τ* is incremented by 1.

### 2.2. Representation of Chemtainer Content and Fusion Thereof

A graph structure is used to represent substances. The vertices of the graph represent monomers. Each vertex is associated with a label that is an element of *S*′ (i.e., the vertex color) denoting a specific type of monomer. Edges between vertices represent bonds between monomers. In addition to these properties of a colored graph, each vertex contains a tuple of linkers. Edges are associated with these linkers such that the degree of a vertex cannot be greater than its number of linkers. If the degree of the vertex is less than the number of linkers, some linkers are considered to be active, allowing for a reaction.

The ordered nature of the linker tuple allows for a limited preservation of the geometric arrangement of the substance. Two substances are considered equal if and only if their graph representation is isomorphic (i.e., the structure of the substances is equal) and their linker tuples match.

In the event of a chemtainer fusion, the chemtainer resulting from the fusion inherits both sets of chemtainer tags from the original chemtainers. The following cases are to be distinguished when generating the graph of a product substance. (a) The active linkers of both substances do not match. (b) The active linkers of both substances match unambiguously. (c) The active linkers of both substances match ambiguously, such that different product substances may result depending on chance.

**Figure 2:**
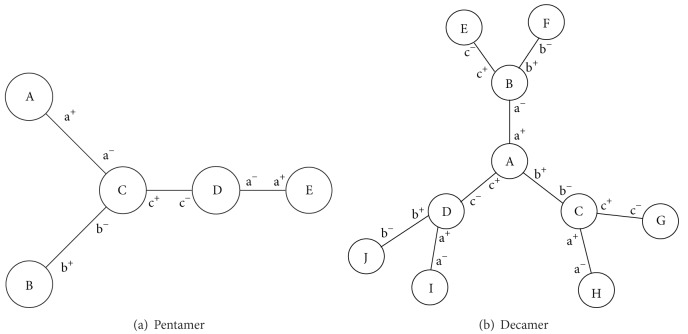
Examples of target polymers. Monomers are denoted by capital letters, and linkers associated with the bonds between monomers are denoted by lowercase letters and a sign; linkers with the same letter and opposite signs are matching linkers.

In case (a), the resulting substance is the graph union of both reactant substances. An example for this case is the reaction of the polymers shown in Figures 2(a) and 2(b). Both these reactants do not have any matching linkers. In fact, they do not have open linkers at all. In case (b), an edge is introduced between the two matching linkers in the graph union. For example, consider the polymer shown in Figure 2(a) and remove the edge between D and E. The resulting two reactants each have one exposed linker, a− and a+, respectively. A reaction of these two reactants thus leads to the original polymer. Note that there is no ambiguity; the only site the linker a− can bind to is the exposed linker a+. Case (c) however might be a source of waste because the substances may react to a product undesired for the synthesis. For example, consider the larger of the two reactants in the previous example, that is, the one consisting of the monomers A, B, C, and D (but not E). Following the same procedure and removing the edge between A and C result in two reactants that can form different products. Particularly The monomer A can bind not only to C but also to D. Since our goal is to maximize yield (i.e., to minimize waste), the algorithm presented in Section 2.3.3 ensures that case (c) is not encountered.

### 2.3. Compiling a Suitable MA for a Given Target Polymer

In this section, we present the steps that are required to find a suitable MA configuration (i.e., cells, tags, and affinities) to synthesize a given target polymer starting from its monomers. The goal is to only allow for steps that have unambiguously matching linkers and to ensure that chemtainer fusion is only possible for pairs of chemtainers in each cell, thus avoiding any waste. Note that we first need to identify the proper sequential chemical pathway at which point we can implement this pathway by configuring the MA appropriately.

Thus, this is a two-step process. First, the possible paths of synthesis are identified. Second, one of these paths is chosen and the MA configuration is derived. Due to the huge number of synthesis paths, the first step is time consuming and requires a lot of storage space. We show at the end of this section that a suitable path of synthesis can be found without enumerating all possible options.

#### 2.3.1. Identifying Paths of Synthesis

Algorithm 1 generates possible paths of synthesis in a recursive top-down approach. The result is a tree of *reaction nodes* and *edge-split nodes*. Reaction nodes are used to estimate the quality of a particular reaction, and edge-split nodes are used to track the edge that is to be connected during the synthesis.

First, a root node is created and the recursion is started with the assembled target polymer as input graph (lines 2–4). One edge *e* = (*v*_1_, *v*_2_) is removed from this input graph (lines 8–9), resulting in two disconnected subgraphs *g*_1_ and *g*_2_ representing two intermediate products. In general, there is no guarantee that the removal of an edge will disconnect the graph; for example, removing the first edge from a cyclic structure will lead to an acyclic, connected graph. In the case of branched polymers however, disconnected sub-graphs can be assumed by definition. The probability *p* for a successful synthesis of the target polymer given the two sub-graphs is computed (line 10). A new reaction node is added as child of the current node in the tree and labelled with *p*. Furthermore, two edge-split nodes are added to the newly inserted reaction node and labelled with *v*_1_ and *v*_2_, respectively (lines 11–13).

The same procedure is repeated recursively with each of the sub-graphs until no more edges can be removed, meaning that the polymer was decomposed to one of its monomers. On each level of the recursion, every edge of the respective input graph is removed as described above.

The runtime of this brute force algorithm scales factorial with the size of the target structure, which is worse than exponential. The runtime could be improved by dynamic programming techniques, where results of the recursion for sub-graphs are memorized in order to avoid multiple computations for the same structure. We have not taken this route, however, as we will introduce an additional constraint in the next section that will prune the search space such that the algorithm performs in linear time.

#### 2.3.2. Selecting Suitable Path of Synthesis

A suitable path can be selected by walking the tree resulting from the algorithm in a manner that never adds a node with *p* < 1 to the fringe. This approach fails if no solution with 100% yield exists. In the case of branched polymers however, this case can be ruled out if the number of distinct linker pairs is at least as large as the degree of any vertex. This allows us to impose the constraint that the linkers associated with each vertex shall be unique. Under this constraint, we prove our claim as follows.

Let us start with the graph of the target polymer, *P*_0_, and remove an arbitrary edge. This leads to two disconnected sub-graphs *P*_1_ and *P*_2_, each of which has one active linker. Because there is only one active linker per subgraph, mixing the substances represented by the two sub-graphs would result in the unambiguous synthesis of the target polymer. Without loss of generality, let us now continue with the first of the two sub-graphs, *P*_1_, and remove an edge from the vertex with the active linker. This decomposition leads to two sub-graphs *P*_3_ and *P*_4_. As before, *P*_1_ can be synthesized by mixing *P*_3_ and *P*_4_ because the newly exposed active linkers are different from the previously exposed ones due to the constraint. Moreover, we can ensure that neither *P*_3_ nor *P*_4_ can react with *P*_1_ by isolating subreactions in different chemtainers. In general, the repeated application of this procedure eventually leads to a monomer which cannot be decomposed any further. Because the constraint ensures that each decomposition can be uniquely synthesized by mixing the substances represented by the resulting sub-graphs, *p* = 1 is guaranteed for each step.

#### 2.3.3. Configuration of the MA

Once a suitable path of synthesis is chosen, the MA is configured. A suitable configuration results in the synthesis of the target polymer from monomers and can be found as follows. First, the reaction nodes of the selected path are visited in a breadth-first manner. This means that the tree is visited level by level, starting from the target polymer at the root, visiting the reactions of the intermediate products. For each node that is visited, a cell is created. Because the root represents the last step of the synthesis, the first node that is visited corresponds to the last cell of the tube and the last node that is visited corresponds to the first cell of the tube. The *m* cells created in that manner are equipped with a unique cell tag.

Second, all monomers of the target polymer are filled into chemtainers. If the molecular graph corresponding to the target polymer consists of *n* nodes, *n* chemtainers are created, each containing the respective monomer. Note that two chemtainers are created if two nodes with a particular color are present. The *n* chemtainers created in this manner are equipped with a unique chemtainer tag.

Third, the affinities are defined. As a consequence of the breadth-first traversal, the first cell corresponds to a reaction node that has two external children. These two children correspond to two chemtainers *c*_*i*_ and *c*_*j*_ containing monomers, which are to be staged inside the inlet. Hence, a positive affinity *a*(*t*_*i*_, *t*_*j*_) > 0 is defined for the interaction between the two tags of the chemtainers *c*_*i*_ and *c*_*j*_. Furthermore, *c*_*i*_ and *c*_*j*_ shall stick to the first cell, and therefore an affinity *a* for the interaction between the cell tag of cell 1 and the tag of chemtainer *c*_*i*_ is defined. The same affinity is also defined for the interaction between the cell tag and the tag of *c*_*j*_, such that (a) both chemtainers stick together and (b) both chemtainers stick to cell 1, thus satisfying the requirements for the fusion of *c*_*i*_ and *c*_*j*_ in the first cell. The selection of a suitable value for *a* is discussed below.

In general, a cell is responsible for either (a) the fusion of chemtainers containing a monomer each, (b) the fusion of a chemtainer containing a monomer with a chemtainer containing an intermediate product, or (c) two chemtainers containing intermediate products each. If a chemtainer contains a monomer, its tag is known because it was assigned while staging the inlet and can therefore readily be used as discussed above. If a chemtainer contains an intermediate product, it is the result of a chemtainer fusion since all original chemtainers were containing monomers. As a consequence, such a chemtainer will carry each of the tags of the fused original chemtainers and a tag can be found by a lookup in the subtree of the corresponding reaction node.

The following considerations are made to compute the affinity of chemtainer-cell interaction *a*. If *a* is too small, the counter *μ*_*i*_ of a chemtainer waiting for fusion with another chemtainer may elapse before the other chemtainer reaches the cell. Therefore, the rule

(2) ai=n+∑j=1i−1aj

is suggested for any affinity *a*_*i*_ involving the cell tag of the *i*th cell. The rule takes into account the release of the *n* chemtainers from the inlet, as well as the maximal time a chemtainer could spend sticking in previous cells.

To summarize, the procedure described above ensures that (a) there is a bijection between reaction nodes and cells, (b) the reactions occur in the proper order, (c) the proper conditions with respect to tag affinity are created for successful chemtainer fusion and subsequent reaction of chemtainer content, and (d) chemtainer fusion does not occur in the absence of a planned reaction.

## 3. Results and Discussion

**Figure 3:**
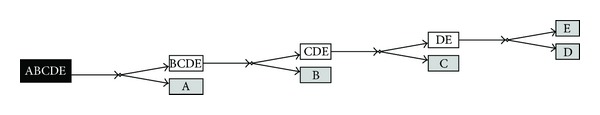
Example of a path of synthesis by sequential insertion for the target polymer shown in Figure 2(a). Edge-split nodes are drawn as rectangles and reaction nodes as small diamonds. The black rectangle denotes the target polymer, white rectangles denote intermediate products, and gray rectangles are monomers. The nodes are added to the tree from left to right as the algorithm progresses. Because the algorithm starts at the target polymer, decomposing it into its monomers, the tree shows the reverse of a synthesis.

The strategy to find all paths of synthesis( recall Algorithm 1) and to configure the MA was implemented and a simulator for the MA was developed. A path of synthesis for the target polymer in Figure 2(a), as computed by Algorithm 1, is displayed in Figure 3. Note that any path of synthesis is a tree by itself. The one shown in the figure is equivalent to a synthesis by sequential insertion because at least one of the two children of each reaction node is a monomer. Although not investigated further in this paper, it did not escape our attention that such a “bushy” path of synthesis would allow the synthesis of several intermediate products in parallel, leading to faster completion of the target polymer.

We also applied our algorithm to the decamer in Figure 2(b) in which case no path of synthesis by sequential insertion without loss (i.e., *p* < 1) was found. This was expected because the polymer was carefully designed to demonstrate that some polymers cannot be synthesized if the number of available linkers is restricted. However the polymer satisfies the constraint of distinct linkers per monomer, and hence our algorithm was able to derive a path of synthesis that does not result in any waste and was also able to compute a configuration of the MA that led to the successful synthesis in simulation.

Our work shows the advantages of compartmentalization for the synthesis of branched polymers. If the number of linkers is limited, we can suppress unwanted reactions by spatially separating the substances. A disadvantage of our method is that Algorithm 1 is computationally heavy and requires a lot of memory as it lays out the whole tree of all possible paths of synthesis. This allows us to find the path which leads to maximal yield by multiplying the probabilities *p* as we traverse the tree, but in the case of branched polymers we know that there exists a path for which *p* = 1 holds at every step as long as the constraint of distinct linkers per vertex is met. Hence, building the full tree is not necessary. Instead, we can simply remove an arbitrary edge from the target polymer and continue to remove edges from the same vertices as discussed in Section 2.3.2. This leads to a path of synthesis with maximal yield in linear time.

The representation of substances based on a graph entails some limitations. First, the substances are assumed to be planar. Second, this representation fails to address the selective synthesis of stereoisomers and similar structures. We note however that the substances could be represented in any way as long as products can be split into reactants, and the probability of a successful synthesis of the product out of these reactants can be estimated. This includes extensions of the graph structure used in this paper as well as even physical simulations.

The MA is modeled as a deterministic device. An according stochastic structure has been studied by B. Reller and presented in [22]. Reller proposed, also *in silico*, a self-assembling microreactor, a two-dimensional structure composed of chemtainers that mediate different linking reactions. This microreactor constitutes a spatially heterogeneous reaction environment which, by its spatial structure, controls or at least amplifies desired reaction pathways. In contrast to the MA, no compiler could be given for Reller's approach.

## 4. Conclusion

In this paper, we have presented a mathematical framework to optimize chemical reactions of branched polymers at high yields by means of compartmentalization. It is emphasized that today no complete implementation of an MA exists. However, MATCHIT delivered important technologies towards an MA. As already mentioned in Section 1 (see references given there), candidates for chemtainers investigated in MATCHIT are vesicles and oil droplets (in water) for which a range of functionalities relevant to the implementation of an MA have been demonstrated. Together with novel types of MEMS devices also resulting from MATCHIT [15], a physical implementation of an MA may be within reach in the near future.

Why may one be interested in the construction of such a system instead of using a series of test tubes in a lab? Besides aspects of automation, there is another, fundamental rationale for investigating designs with the potential for miniaturization [22]. We hypothesize that miniaturized version of the MATCHIT automaton could provide opportunities to use types of catalysts not used in conventional chemical process management. Assume that one synthesizes oligomers with some sort of catalytic activity, catalytic activity that is used in a subsequent step. It may well be the case that the environment in which this catalyst is synthesized (or activated) is different from the environment in which the catalyst has to act. In a macroscopic laboratory, this implies that the catalyst has to be transferred from one environment to the other, a process that takes time. This means that catalysts have to fulfill two requirements: first, they have to be efficient, and, second, they have to be stable. On a microscopic length scale, where transport involves transport over micrometers, ten seconds is quite a time (even if this transport happens by diffusion). This means that in microscopic reactors, catalysts, if they are produced *in situ*, only have to be efficient but no longer need to be particularly stable. An average life time of minutes to fractions of a second is sufficient for typical lengths ranging from several ten *μ*m down to part of *μ*m, depending on passive diffusion and active processes such as electrical fields [15]. As a result, many more molecules qualify as potential catalysts. The possibility that microreactors could enable the use of metastable catalysts in a technically feasible manner is a more than sufficient justification for the study of such systems.

## Figures and Tables

**Algorithm 1:**
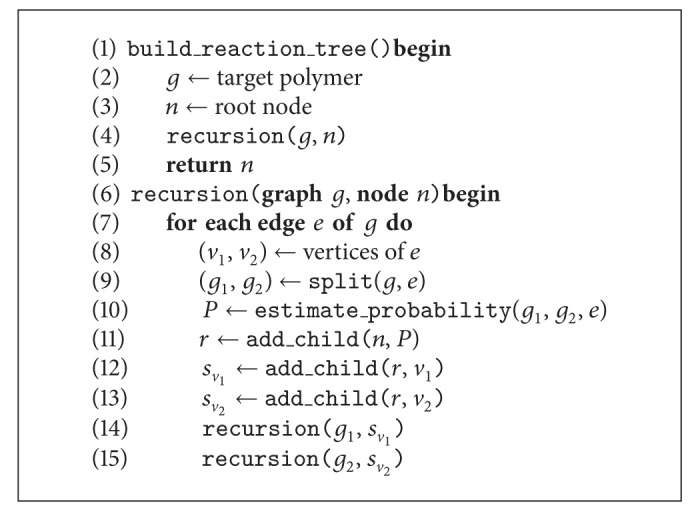
Algorithm to recursively find possible paths of synthesis for a target polymer.

## References

[1] http://fp7-matchit.eu.

[2] Amos M, Dittrich P, McCaskill J, Rasmussen S Biological and chemical information technologies.

[3] Symes MD, Kitson PJ, Yan J (2012). Integrated 3D-printed reactionware for chemical synthesis and analysis. *Nature Chemistry*.

[4] Villar G, Graham AD, Bayley H (2013). A tissue-like printed material. *Science*.

[5] Seelig G, Soloveichik D, Zhang DY, Winfree E (2006). Enzyme-free nucleic acid logic circuits. *Science*.

[6] Zhang DY, Winfree E (2009). Control of DNA strand displacement kinetics using toehold exchange. *Journal of the American Chemical Society*.

[7] Qian L, Winfree E (2011). Scaling up digital circuit computation with DNA strand displacement cascades. *Science*.

[8] van Swaay D, deMello A (2013). Microuidic methods for forming liposomes. *Lab on a Chip*.

[9] Hadorn M, Eggenberger Hotz P (2010). DNA-mediated self-assembly of artificial vesicles. *PloS ONE*.

[10] Hadorn M, Boenzli E, Sffrensen KT, Fellermann H, Hotz PE, Hanczyc MM (2012). Speciffc and reversible DNA directed self-assembly of oil-in-water emulsion droplets. *Proceedings of the National Academy of Sciences*.

[11] Caschera F, Rasmussen S, Hanczyc MM (2013). An oil droplet division-fusion cycle. *ChemPlusChem*.

[12] Sunami T, Caschera F, Morita Y (2010). Detection of association and fusion of giant vesicles using a fluorescence-activated cell sorter. *Langmuir*.

[13] Caschera F, Sunami T, Matsuura T, Suzuki H, Hanczyc MM, Yomo T (2011). Programmed vesicle fusion triggers gene expression. *Langmuir*.

[14] Hadorn M, Boenzli E, Hotz PE, Hanczyc MM (2012). Hierarchical unilamellar vesicles of controlled compositional heterogeneity. *PloS One*.

[15] Wagler PF, Tangen U, Maeke T, McCaskill JS (2012). Field programmable chemistry: integrated chemical and electronic processing of informational molecules towards electronic chemical cells. *BioSystems*.

[16] Cummings RD (2009). The repertoire of glycan determinants in the human glycome. *Molecular BioSystems*.

[17] Varki A (1993). Biological roles of oligosaccharides: all of the theories are correct. *Glycobiology*.

[18] Koeller KM, Wong C-H (2000). Complex carbohydrate synthesis tools for glycobiologists: enzyme-based approach and programmable one-pot strategies. *Glycobiology*.

[19] Kolb HC, Finn MG, Sharpless KB (2001). Click chemistry: diverse chemical function from a few good reactions. *Angewandte Chemie—International Edition*.

[20] Kolb HC, Sharpless KB (2003). The growing impact of click chemistry on drug discovery. *Drug Discovery Today*.

[21] Best MD (2009). Click chemistry and bioorthogonal reactions: unprecedented selectivity in the labeling of biological molecules. *Biochemistry*.

[22] Füchslin RM, Dzyakanchuk A, Flumini D (2013). Morphological computation and morphological control: steps toward a formal theory and applications. *Artiffcial Life*.

[23] Dittrich P, Ziegler J, Banzhaf W (2001). Artificial chemistries—a review. *Artificial Life*.

[24] http://www.cobra-project.eu.

